# Neuroprotective Effects of Cilomilast and Chlorogenic Acid Against Scopolamine-Induced Memory Deficits via Modulation of the cAMP/PKA–CREB–BDNF Pathway

**DOI:** 10.3390/ijms26073108

**Published:** 2025-03-28

**Authors:** Esraa M. Mosalam, Soha M. Atya, Noha M. Mesbah, Shady Allam, Eman T. Mehanna

**Affiliations:** 1 Biochemistry Department, Faculty of Pharmacy, Menoufia University, Shebin EL-Kom 32511, Menoufia, Egypt; 2 Department of Pharm D, Faculty of Pharmacy, Jadara University, Irbid 21110, Jordan; 3 Department of Biochemistry, Faculty of Pharmacy, Suez Canal University, Ismailia 41522, Ismailia, Egypt; pgs.14220574@pharm.suez.edu.eg (S.M.A.); noha_mesbah@pharm.suez.edu.eg (N.M.M.); 4 El-Bagour Specialized Hospital, El-Bagour 32821, Menoufia, Egypt; 5 Department of Pharmacology and Toxicology, Faculty of Pharmacy, Menoufia University, Shebin EL-Kom 32511, Menoufia, Egypt; shady_allam@phrm.menofia.edu.eg

**Keywords:** Alzheimer’s, cilomilast, chlorogenic acid, phosphodiesterase 4, cAMP/PKA–CREB–BDNF pathway, neuroinflammation

## Abstract

Alzheimer’s disease (AD) is a neurodegenerative disorder characterized by cognitive decline, neuroinflammation and neuronal damage. This study aimed to investigate the neuroprotective effects of cilomilast (CILO), a phosphodiesterase-4 (PDE4) inhibitor, alone and in combination with chlorogenic acid (CGA), a natural polyphenol, against scopolamine (SCOP)-induced cognitive impairment in mice. Forty male albino mice were divided into five groups: normal control, SCOP control, CGA + SCOP, CILO + SCOP and CILO + CGA + SCOP. Behavioral assessments, including the Y-maze and pole climbing tests, demonstrated that SCOP significantly impaired cognition, while treatment with CILO and CGA reversed these deficits, with the combination group showing the greatest improvement. Histopathological analyses revealed that CILO and CGA reduced neuronal damage and amyloid beta (Aβ) accumulation. Immunohistochemical and biochemical assessments confirmed a decrease in neuroinflammatory markers, including tumor necrosis factor-alpha (TNF-α) and nuclear factor kappa B (NF-κB). Molecular analyses showed that CILO restored cyclic adenosine monophosphate (cAMP) levels, leading to activation of protein kinase A (PKA), cAMP response element-binding protein (CREB) and brain-derived neurotrophic factor (BDNF), key regulators of neuronal plasticity and survival. CGA enhanced these effects by further inhibiting PDE4, amplifying the neuroprotective response. These findings suggest that PDE4 inhibitors, particularly in combination with CGA, may represent promising therapeutic strategies for AD-related cognitive impairment.

## 1. Introduction

Alzheimer’s disease (AD) is a neurodegenerative disorder marked by a permanent progressive loss of neuronal cells, which in turn impairs memory, causes cognitive dysfunction and eventually diminishes the patient’s capacity to perform everyday duties [1]. Currently, more than 50 million people worldwide suffer from dementia. This number is expected to be duplicated every 20 years, rising to 82 million by 2030 and 152 million by 2050 [2]. Presently, AD is the most predominant type of dementia, comprising 60–70% of all cases of dementia [3].

The pathogenic hallmarks of AD involve, but are not limited to, accumulated amyloid beta (Aβ) and phosphorylated tau protein that result in a buildup of neurotic plaques and neurofibrillary tangles (NFTs), respectively [4]. AD is also characterized by increased oxidative stress, decreased cholinergic neurotransmission, downregulated cyclic adenosine monophosphate (cAMP)-response element-binding protein (CREB) and flaring inflammatory response [5].

Several biological agents including neuromodulators, intracellular elements, plant extracts and nutritional components, may promote neurotransmission and enhance the cognitive performance [6]. Targeting the phosphodiesterase-4 (PDE4) enzyme may be crucial in reversing memory deterioration linked to aging and AD [7]. PDE4 inhibitors can act as neuroprotective agents through enhancing neuronal recovery, neuronal plasticity, long-term potentiation and neurogenesis [8]. This is because the PDE4 isoenzyme is a crucial regulator of intracellular cAMP that is significantly expressed in the hippocampus and the frontal cortex [9]. Accumulation of intracellular cAMP, caused by PDE4 inhibition, triggers the phosphorylation of CREB by protein kinase A (PKA), leading to subsequent upregulation of the brain-derived neurotrophic factor (BDNF) [10]. Cognitive deficiencies in AD are thought to be linked to the downregulation of CREB, a critical transcriptional factor for both learning and memory [11]. Decreased CREB phosphorylation in the case of AD is caused by Aβ buildup through a decrease in the level of cAMP and changes in the structure of the regulatory and/or catalytic subunits of PKA [12].

Cilomilast (CILO) is a potent and specific PDE4 inhibitor. It acts by a mechanism similar to roflumilast with a more significant anti-inflammatory action and fewer side effects owing to its greater selectivity towards PDE4 [13]. Roflumilast has been reported to improve AD manifestations by elevating the levels of cAMP, CREB and BDNF [14]. It also acts by lowering the level of proinflammatory cytokines such as tumor necrosis factor-alpha (TNF-α), interferon-γ (IFN-γ), nuclear factor kappa B (NF-κB) and interleukin-17 (IL-17) [15]. However, most of the PDE inhibitors have dose-limiting adverse effects including nausea, vomiting and stomach acidity, which led to limited clinical compliance [16]; this is an issue that may be defeated through combination with other therapeutics to enhance the effectiveness and decrease the unpleasant side effects.

Chlorogenic acid (CGA), an ester of quinic and caffeic acids, is a significant polyphenol found in *Coffea canephora*, *Coffea arabica* L. and *Mate* (*Ilex paraguariensis*). The weight of CGA in green coffee beans ranges from 5 to 12 percent. According to the clinical research, drinking coffee with excessive amounts of CGA reduced mental weariness and headaches, showed positive impacts on mood and slowed down the aging process [17]. There is an evidence that CGA has anti-oxidative, anti-inflammatory, neuroprotective, neurotrophic and neuro-nutrition properties [18]. CGA also enhanced spatial learning and memory besides extending the lifespan of dopaminergic neurons [19].

In the current work, we assumed that combining the natural polyphenolic CGA with CILO may augment its neuroprotective effect. Consequently, our study aims to investigate the neuroprotective effect of CILO and/or CGA against a scopolamine (SCOP)-induced classic AD-like amnesic model in mice, focusing on the cAMP/CREB/BDNF signaling pathway. To the best of our knowledge, this is the first study to investigate the neuroprotective efficacy of CILO against SCOP-induced AD-like models in mice, as well as to explore the potential enhancement of this effect through its combination with CGA.

## 2. Results

### 2.1. Effect on Behavior

**Figure 1:**
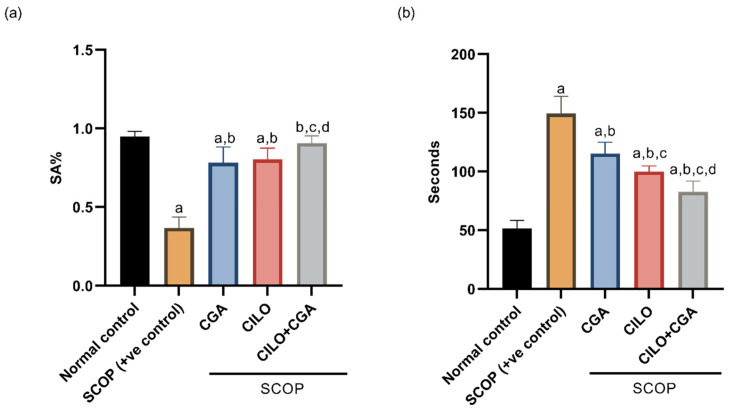
Effect on behavioral tests. (**a**) Y-maze test and (**b**) pole climbing test. Data are displayed as the mean ± SD, *n* = 8, and were evaluated using one-way ANOVA and Tukey post hoc analysis, *p* < 0.05. a: significant versus normal control, b: significant versus SCOP group, c: significant versus CGA group and d: significant versus CILO group. SCOP: scopolamine, CGA: chlorogenic acid, CILO: cilomilast, SA%: percentage of spontaneous alteration.

Figure 1a displays the percent of spontaneous alteration (SA%) of the Y-maze test on day 7. The sole injection of SCOP significantly decreased SA% by 61.3% (*p* < 0.001) when compared to the control group. The CGA group showed a significant (*p* < 0.001) increase in SA% relative to the SCOP group. Similarly, the SA% was significantly (*p* < 0.001) increased by the CILO pretreatment relative to the SCOP group. The pretreatment with CILO + CGA in the combination group boosted the SA% significantly by 1.5-fold (*p* < 0.001) compared with the SCOP group, indicating that spatial memory was remarkably enhanced by CILO and/or CGA pretreatment.

Regarding the pole climbing test, the results are represented in Figure 1b. SCOP-model mice exhibited a significant longer time to turn their heads and go down (1.9-fold, *p* < 0.001) relative to the normal control group. In contrast, CGA-pretreated mice showed significantly less time compared to SCOP control mice. In the same manner, CILO-pretreated mice showed a significant (*p* < 0.001) reduction in the latency to descend the pole compared to the positive control mice. Notably, mice in the CILO + CGA combination group significantly spent the shortest time to climb and reach the ground in comparison with the SCOP mice (44.6%, *p* < 0.001).

### 2.2. Histopathological Findings

**Figure 2:**
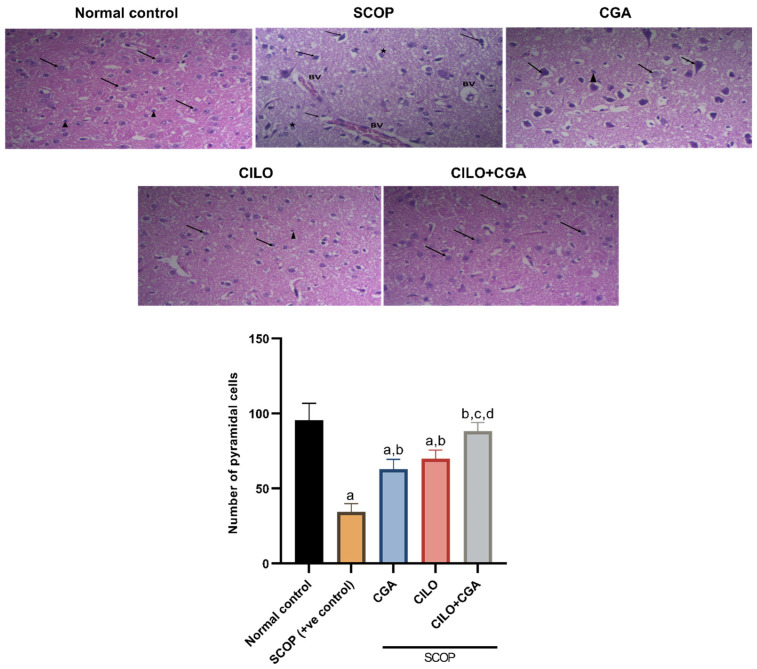
Photomicrographs of brain tissue stained with hematoxylin and eosin (H & E). The scale bar is 50 μm, and the magnification is 200×. Results are expressed as the mean ± S.D, *n* = 4. Statistical analysis was carried out by one-way ANOVA followed by the Tukey post hoc test. Values were considered significantly different at *p* < 0.05. a: significant versus normal control, b: significant versus SCOP group, c: significant versus CGA, d: significant versus CILO. SCOP: scopolamine, CGA: chlorogenic acid, CILO: cilomilast. Black arrow: pyramidal cell, arrowhead: few astrocytes, BV: blood vessels, star: multiple astrocytes.

Figure 2 shows the histopathological findings after hematoxylin and eosin (H & E) staining of the brain tissue of experimental mice. Microscopical examination of the sections isolated from the normal control group revealed normal pyramidal cells of the brain (arrow) and some astrocytes (arrowhead). In contrast, the photomicrographs of the SCOP group revealed several histological abnormalities; pyramidal cells were severely damaged, appearing shrunken and irregular in shape, with hyperchromatic nuclei eosinophilic to vacuolated cytoplasm (arrows). Additionally, dilated blood vessels (BVs) were seen congested and surrounded by inflammation. In the CGA-pretreated group, moderate improvement was noticed in the brain tissue, which showed nearly normal pyramidal and granular cells (arrow), also with a few astrocytes (arrowhead). Similarly, the photomicrographs of the CILO group revealed typical granular and pyramidal cells (arrow), and few astrocytes were also observed (arrowhead). The highest neuroprotection was observed in the CILO + CGA combination group, which showed a likely normal architecture of the examined brain tissue where the pyramidal cells appeared with vesicular rounded nuclei (arrow).

The quantitative analysis of the pyramidal cells in the SCOP positive control mice showed a significant loss of the pyramidal cells (64%, *p* < 0.001) relative to the normal control mice. Pretreatment of the mice with either CILO or CGA showed a significant rise in the number of pyramidal cells compared to the SCOP group. The most significant increase in the number of pyramidal cells was observed in the combination group by 1.6-fold (*p* < 0.001), as illustrated in Figure 2.

**Figure 3:**
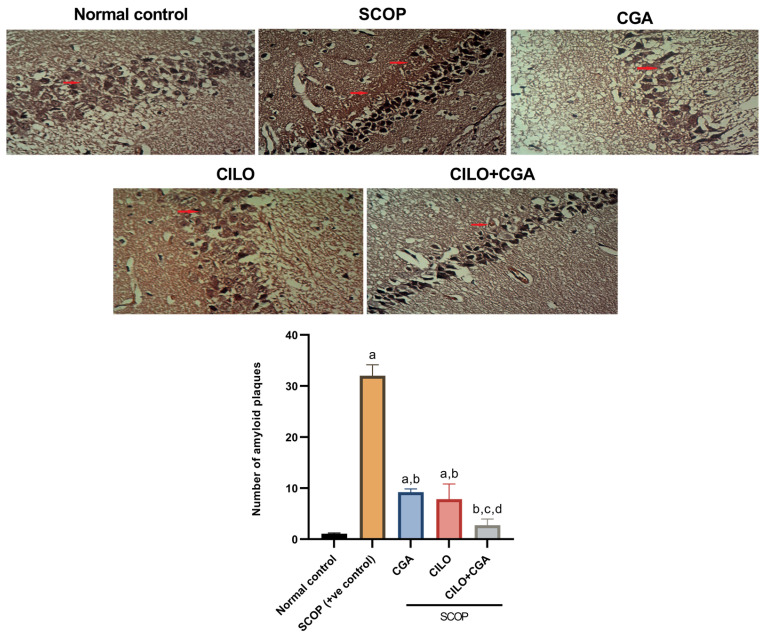
Photomicrographs of brain regions stained with Congo red. The scale bar is 50 μm and the magnification is 200×. Results are expressed as the mean ± SD, *n* = 4. Statistical analysis was performed using one-way ANOVA followed by the Tukey post hoc test. Values were considered significantly different at *p* < 0.05. a: significant versus normal control, b: significant versus scopolamine c: significant versus CGA, d: significant versus CILO. SCOP: scopolamine, CGA: chlorogenic acid, CILO: cilomilast. Red arrow: amyloid plaque deposition.

Figure 3 represents Congo red staining for visualization of Aβ deposits. The photomicrographs of the SCOP group revealed multifocal strong deposition of Aβ plaques in the examined brain tissue. Moderate deposition of Aβ plaques was shown in the CGA-pretreatment group. Mice pretreated with CILO showed mild to moderate congophilic amyloid plaques. The CILO + CGA combination group exhibited mild to minimal reactivity toward the Congo red staining and more decreased plaque deposition in the examined brain tissue. Analysis of the photomicrographs revealed that the visualized Aβ deposits were increased significantly (28.8 times, *p* < 0.001) in the SCOP group compared to the normal control group. In contrast, each of CILO and CGA separately significantly decreased the amyloidosis in comparison with the SCOP control group as displayed in Figure 3. The co-pretreatment with CILO + CGA remarkably decreased the congophilic amyloid plaques by 91.4% (*p* < 0.001) compared to the SCOP group.

### 2.3. Effect on Inflammatory Mediators

**Figure 4:**
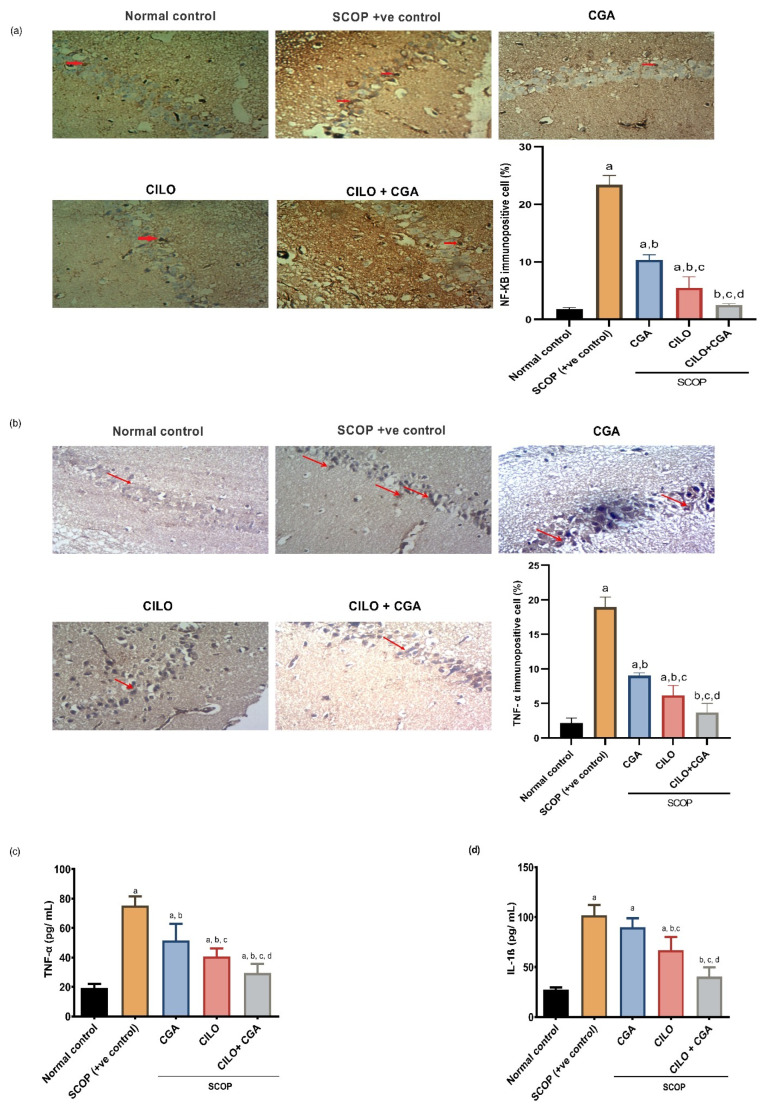
(**a**) Immunohistochemical analysis of NF-κB expression, (**b**) immunohistochemical analysis of TNF-α expression, (**c**) effect on brain content of TNF-α and (**d**) effect on brain content of IL-1β. The scale bar is 50 μm and the magnification is 200×. Data are expressed as mean ± SD, *n* = 4 for the immunohistochemical analyses (**a**,**b**) and *n* = 8 for the ELISA analyses (**c**,**d**). The data were analyzed using one-way ANOVA followed by the Tukey post hoc test, *p* < 0.05. a: significant versus normal control group, b: significant versus SCOP group, c: significant versus CGA, d: significant versus CILO. SCOP: scopolamine, CGA: chlorogenic acid, CILO: cilomilast, NF-κB: nuclear factor kappa B, TNF-α: tumor necrosis factor-alpha, IL-1β: interleukin 1β. The red arrows in (**a**) represent NF-κB immunopositive cells, whereas in (**b**) represent TNF-α immunopositive cells.

Immunohistochemistry (IHC) findings revealed that the SCOP group showed high reactivity toward anti-NF-κB and anti-TNF-α. The morphometric analysis showed that SCOP increased the expression of NF-κB and TNF-α by 12.2- and 7.8-fold (*p* < 0.001), respectively, in comparison with the normal control group, as demonstrated in Figure 4a,b. Contrarily, the mice that were pretreated with each of CILO or CGA showed moderate reactivity toward the antibodies, but the CILO group exhibited significantly lower expression of those inflammatory mediators compared to CGA when assessed morphometrically. Co-administration of CILO and CGA exhibited mild reactivity toward anti-NF-κB and anti-TNF-α. By quantitative means, the combination group significantly (*p* < 0.001) reduced the NF-κB- and TNF-α-immuno-positive cells by 89.2% and 80.5%, respectively, when compared to the SCOP group.

To provide further proof for the effect of the investigated pretreatments on neuroinflammation, the levels of TNF-α and interleukin-1β (IL-1β) were also determined in the brain tissue by Enzyme-linked Immunosorbent Assay (ELISA). Both TNF-α and IL-1β in the SCOP group were significantly (*p* < 0.001) higher in comparison with the normal control by 2.9- and 2.7-fold, respectively. The CGA alone showed a significant reduction in TNF-α, but there was no significant change in the concentration of IL-1β (*p* = 0.117) against the SCOP group. Compared to the SCOP group, the CILO group exhibited a significant (*p* < 0.001) decrease in the levels of TNF-α and IL-1β. Administration of CILO + CGA in combination showed a significant (*p* < 0.001) reduction in both cytokines by 60.7 and 60.4%, respectively, relatively to the SCOP positive control mice (Figure 4c,d).

### 2.4. Effect on cAMP, PKA, CREB, and BDNF

**Figure 5:**
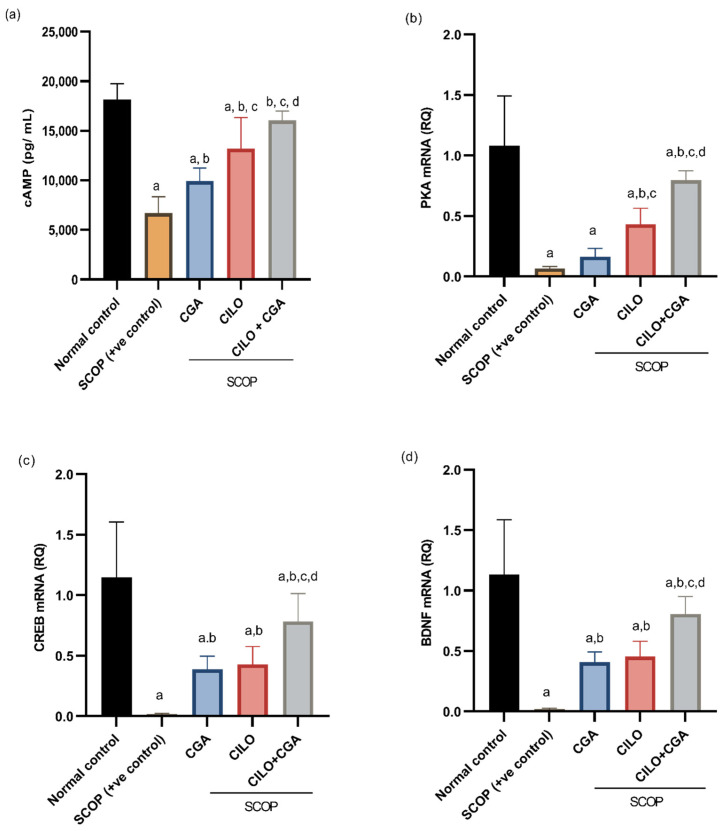
Effect on the cAMP/PKA–CREB–BDNF pathway. (**a**) The brain content of cAMP, (**b**) expression level of PKA, (**c**) expression level of CREB and (**d**) expression level of BDNF. Data are presented as the mean ± SD, *n* = 8 and were analyzed using one-way ANOVA followed by the Tukey post hoc test, *p* < 0.05. a: significant versus normal control, b: significant versus SCOP group, c: significant versus CGA, d: significant versus CILO. SCOP: scopolamine, CGA: chlorogenic acid, CILO: cilomilast, cAMP: cyclic adenosine monophosphate, PKA: protein kinase A, CREB: cAMP response element-binding protein, BDNF: brain-derived neurotrophic factor.

The level of cAMP in the brain tissue of the SCOP group was significantly (*p* < 0.001) decreased compared to the control group by 63%. On the contrary, the level of cAMP was significantly reversed in the CILO- and CGA-pretreated groups compared to the SCOP group, but CILO showed a more significant increase than CGA. The co-administration of CILO + CGA has boosted the concentration of cAMP in the brain relative to the SCOP group by 1.4-fold (*p* < 0.001), as shown in Figure 5a.

On the other hand, the quantitative real-time polymerase chain reaction (qRT-PCR) results indicated that injection of SCOP significantly (*p* < 0.001) induced a decline in the gene expression of PKA, CREB and BDNF in the brain tissues of the mice by 93.8, 98.5 and 98.2%, respectively, compared to the normal control. The mice that were protected by CILO or CGA showed significant upregulation of these genes, except for the PKA gene in the CGA group, which showed a non-significant increase (*p* = 0.931) relative to the SCOP group. In the CILO + CGA combination group, the mice showed significant upregulation of PKA, CREB and BDNF by 10.9-, 45.7- and 39-fold (*p* < 0.001), respectively, when compared with the SCOP group (Figure 5b–d).

### 2.5. Effect on PDE4

**Figure 6:**
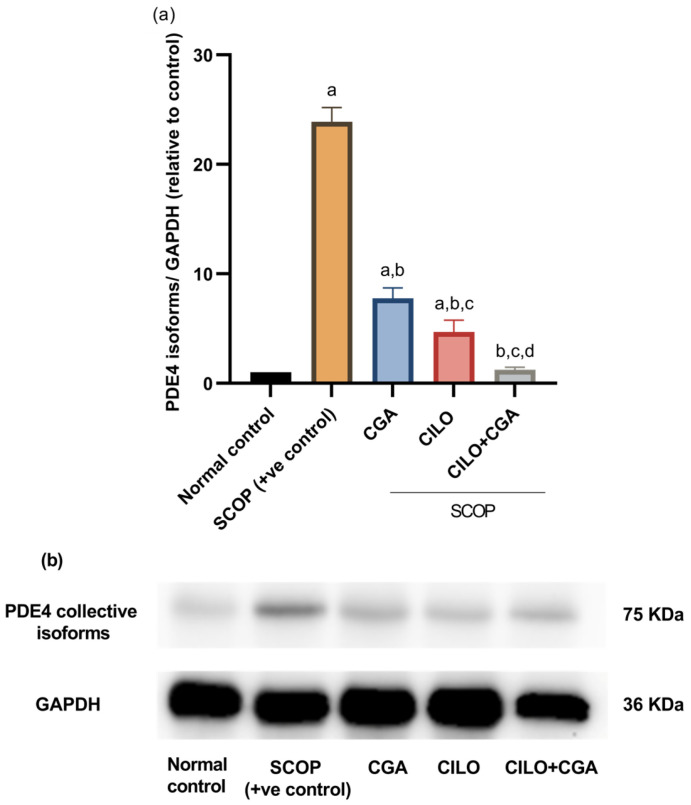
Western blot analysis for collective isoforms of PDE4 in the brain tissue. GAPDH was used as an internal control. (**a**) Normalization relative to normal control and (**b**) protein bands. Values represent the mean ± SD, *n* = 3, and were analyzed using ANOVA (one-way) followed by the Tukey post hoc test, *p* < 0.05. a: significant versus normal control, b: significant versus SCOP group, c: significant versus CGA, d: significant versus CILO. SCOP: scopolamine, CGA: chlorogenic acid, CILO: cilomilast, PDE4: phosphodiesterase 4.

In order to ascertain the mechanism by which CILO protects the brain, Western blotting was utilized to quantify PDE4 subtype proteins collectively. The findings indicated that a significant upregulation of PDE4 collective isoform expression (*p* < 0.001) was observed in the SCOP-treated mice compared with the normal control mice. The effect was significantly reversed in the CILO- or CGA-pretreated groups. However, administration of CILO in combination with CGA resulted in the most significant (*p* < 0.001) decrease in PDE4 total expression against the SCOP-model mice, as shown in Figure 6.

### 2.6. Protein–Protein Network Analysis

**Figure 7:**
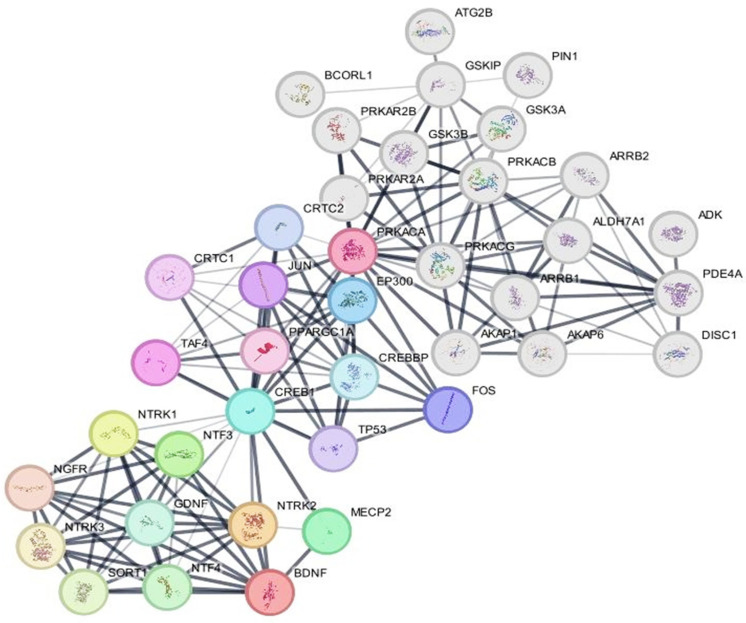
Protein–protein interaction network analysis. The network was generated by the Cytoscape software v3.10.2 based on the STRING database. Nodes represent individual proteins, edges indicate protein–protein interactions and the line thickness reflects confidence scores of interactions. Number of nodes = 39, number of edges = 155, clustering coefficient = 0.764 and confidence score = 0.4. The prominent hub proteins, PKA, CREB, BDNF and PDE4, are the central proteins in this network. The other visualized interacted proteins may act as further therapeutic targets.

PKA, CREB, BDNF and PDE4 were selected as central proteins to create separate networks, followed by a merged network to predict other molecular target proteins and pathways that could be valuable diagnostics or therapeutics, as presented in Figure 7. Nodes represent individual proteins, edges indicate protein–protein interactions, and the line thickness reflects confidence scores of interactions. PKA is denoted by multiple subunits: PRKACA (subunit alpha), PRKACB, PRKACG, PRKAR2A and PRKAR2B. CREB is represented as CREB1 (the principal transcription factor) and CREB-binding protein (CREBBP), both in light blue, indicating their role in transcriptional regulation. BDNF and PDE4 are also represented in a red and grey node, respectively. In more details, the network shows three major distinct cross-talked pathways: cAMP–PKA–CREB, the neurotrophin signaling pathway and PDE4/cAMP. On one hand, the cAMP–PKA–CREB trajectory seems to be the central pathway in this network, in which PKA phosphorylates CREB1, which then recruits CREBBP to activate the transcription of the downstream target genes such as BDNF and Fos proto-oncogene, AP-1 transcription factor subunit (FOS). This pathway is critical for neuronal plasticity and memory formation. On the other hand, BDNF, in the neurotrophin signaling pathway, interacts with neurotrophic receptor tyrosine kinase 2 (NTRK2) with subsequent activation of some intracellular pathways like mitogen-activated protein kinase/extracellular signal-regulated kinase pathway (MAPK/ERK), which further reinforces CREB phosphorylation. Lastly, it is well known that PDE4 principally degrades cAMP, limiting the activity of PKA and thus modifying the PKA–CREB–BDNF axis.

## 3. Discussion

Alzheimer’s disease (AD) is the most prevalent type of senile dementia that significantly impairs patients’ perception, memory, judgment and cognitive abilities [20]. Although the specific mechanisms underlying this illness are still not entirely known, mounting evidence points to the possibility that abnormal accumulation of Aβ as well as phosphorylated tau proteins, neuroinflammation and oxidative stress may all be key factors [21]. As time passes, neurons gradually become affected, impaired and eventually perish [22].

To the best of our knowledge, this work is the first to investigate the effect of CILO against SCOP-induced neurotoxicity. We aimed to examine whether CILO, either alone or in combination with CGA, can effectively reverse the cognitive impairment induced by SCOP. In addition, we focused on the possible involvement of the cAMP/CREB/BDNF pathway in the development of AD, as well as the mechanistic effects of CILO and/or CGA in this context.

In our study, the injection of SCOP not only impaired cognition and spatial memory but also disabled locomotor function and coordination. The effect was illustrated by the apparent neurodegeneration in H & E staining and the amyloidosis in Congo red staining. The elevated neuroinflammatory markers, including NF-κB, TNF-α and IL-1β, together with decreased levels of cAMP, PKA, CREB and BDNF, confirmed the amnesic effect of SCOP in our model group.

This may be because SCOP is a non-selective centrally acting muscarinic receptor antagonist that blocks cholinergic neurotransmission [23], and impairs learning and memory in both rodents and humans [24]. It is widely recognized that SCOP disrupts learning and memory across a range of behavioral assessments due to its dual mechanism of action: inhibition of the cholinergic system and downregulation of the expression of CREB with BDNF [25]. Intraperitoneally injected SCOP can increase reactive oxygen species (ROS) through modulation of antioxidant enzymes, buildup of amyloid plaques, suppression of trophic factors and induction of neuroinflammation that may eventually lead to atrophy and degeneration of the neurons [26]. It has also been demonstrated that SCOP administration increases the pro-inflammatory cytokines, including NF-κB, which simultaneously triggers the activation of cyclooxygenase 2 (COX2) and facilitates its translocation to the nucleus [27]. Therefore, inflammation has been postulated as the main mechanism underlying SCOP-induced cognitive impairment [28]. Aligning with our results, previous works demonstrated that the levels of TNF-α, IL-6 and IL-1β have been increased in rodents’ brains after SCOP injection [29,30,31].

CILO is a potent PDE4 inhibitor that acts by increasing cAMP with subsequent upregulation of the CREB pathway [32]. It has been reported that selective PDE4 inhibitors, including CILO, apremilast and roflumilast, inhibit the inflammatory response both in vitro and in vivo [33]. In our study, CILO pretreatment significantly amended the learning and memory impairments induced by SCOP, as revealed by the behavioral performance of the experimental mice. Moreover, CILO effectively alleviated neuronal injury and inflammation, as indicated by the histopathological and immuno-staining. These effects of CILO were also associated with a restored cAMP/PKA and enhanced CREB/BDNF signaling cascade. These outcomes are in line with a previous work that studied another PDE4 inhibitor, FCPR03 [33]. Consequently, the activated cAMP/PKA/CREB signaling pathway inhibits the release of pro-inflammatory cytokines and demonstrates substantial anti-inflammatory effects.

Several studies have pointed out that cyclic nucleotides are crucial for cognitive functions [34]. cAMP is assumed to be the main positive regulator of the transcriptional, translational and post-transcriptional modifications that are affecting key molecules involved in long-term potentiation (LTP), synaptic plasticity and memory consolidation and retrieval [35,36]. CREB, which is activated by PKA, plays a crucial role in the proper functioning of the central nervous system (CNS), besides neuroprotection against a variety of pathological effectors [5]. Multiple studies have demonstrated that CREB phosphorylation regulates BDNF expression via a variety of pathways, including the mitogen-activated protein kinase (MAPK)/extracellular signal-regulated kinase (ERK) pathway [37]. This suggests a clear correlation between the evolution of cognitive problems and the downregulation of CREB and BDNF [38]. cAMP also has the potential to control the transcription of inflammatory genes by the intracellular sensors, including EPAC 1/2, which triggers the transcription protein Ras-related protein 1 (Rap 1) [39]. Furthermore, PKA targets NF-κB, which promotes the generation of proinflammatory cytokines; thereby, their levels are decreased [40].

BDNF has been recognized to have a major impact on AD through maintaining the survival of neuronal cells, preventing neurodegeneration and managing synaptic plasticity for learning and memory functions [41]. Our study showed a significant reduction of PKA, BDNF and CREB levels following administration of SCOP, which was in line with a previous report that SCOP promoted cognitive deficits in rodents by impairing the CREB–BNDF neurotrophic pathway [42,43]. In contrast, CILO compensated for the downregulation of BDNF after restoring PKA and CREB in SCOP mice.

Interestingly, inhibition of PDE4, a specific enzyme that degrades cAMP [44], has exhibited a great potential for AD treatment through increasing the intracellular level of cAMP [45]. Similarly, it has been confirmed that selective PDE-4D inhibitors like clioquinol and rolipram/roflumilast hybrids have improved memory and cognition in a variety of research studies [46]. A prior study reported that PDE4B-deficient mice displayed antidepressant-like behavior and dramatically boosted neurogenesis [47]. Another study also demonstrated that rolipram and related drugs counteract the amnesic effect of SCOP on memory via enhancing the cAMP level and PKA/CREB signaling cascade [48].

CGA, one of the most readily available polyphenols, activates the PKA/CREB pathway and possesses antioxidant and anti-inflammatory properties [49]. Furthermore, ample studies have proved that CGA can reduce oxidative stress and provide a neuroprotective effect in SCOP-induced amnesia with significant improvement in the impairment of short-term memory or working memory [50]. Injection of mice with CGA in the current study reversed the memory dysfunction in the behavioral paradigms reflecting higher level of cognition and locomotion compared to the SCOP group.

Our histopathological and IHC findings showed that CGA also alleviated the pyramidal cell loss, diminished the Aβ accumulation and suppressed the flare of neuroinflammatory markers induced by SCOP. This was supported by previous studies demonstrating that CGA and its derivatives enhanced the memory and cognitive functions of mice and effectively decreased the buildup of Aβ by promoting the proteasomal degradation of β-secretase [51,52]. A former study has reported that CGA may regulate aberrant microglia activity by blocking the activation of the NF-kB/P38 MAPK inflammatory pathway and decreasing the release of inflammatory cytokines, such as TNF-α and IL-1β [53]. Moreover, it upregulates the expression of CREB and decreases inflammation in the aged brain by inhibiting the NF-κB signaling pathway [54]. Additionally, CGA has shown favorable restoration for cAMP, CREB and BDNF; this effect could explain the neuroprotection in the CGA group that was reflected by the enhanced behavioral performance of the mice. This neuroprotective effect may be due to other kinases’ involvement, as reported in a previous study [55], and to some extent may be a result of the significant downregulation in the total expression of PDE4.

Regarding the effect of CILO + CGA combined therapy, the co-administration of both agents showed superior findings compared to each of them alone. There was a remarkable amendment in the architecture of the brain and less amyloidosis, together with decreased levels of inflammatory cytokines and upregulation of cAMP, PKA, CREB and BDNF, which may result from the augmented inhibition of PDE4. A similar neuroprotective effect was reported previously when CGA was administered in combination with flumazenil as well as diazepam [56] and glutamate [57].

PPI network analysis was also performed to provide more mechanistic perceptions that support our findings. The network analysis revealed the key roles of PKA, CREB, BDNF and PDE4 in neuronal signaling, integrating the cAMP–PKA–CREB and neurotrophin signaling pathways. The network confirms the notion that the modulatory effect of PDE4 on cAMP is crucial and can influence cognitive functions. Therefore, targeting PDE4 and modulation of the cAMP/PKA–CREB–BDNF trajectory may represent a potential therapeutic value in neuroinflammatory disorders including AD.

## 4. Materials and Methods

### 4.1. Drugs and Chemicals

CILO was obtained from Adooq Bioscience (Irvine, CA, USA). CGA was from Cayman Chemical (Ann Arbor, MI, USA). Dimethyl sulfoxide (DMSO) was from Alpha Chem (Gardena, CA, USA). SCOP was from LKT laboratories (St. Paul, MN, USA). Phosphate buffered saline (PBS) was from Serox GmbH (Mannheim, Germany). All other chemicals were of high grade.

### 4.2. Animals

The experimental mice were obtained from The Egyptian Organization for Biological Products and Vaccines (VACSERA, Cairo, Egypt). They were 8–10 weeks old and weighed 30–35 gm. The mice were kept in a plastic acrylic housing for about 2 weeks before the experiment for adaptation, with standard environmental conditions including a 12 h dark/12 h light cycle and a temperature of 23 ± 2 °C. They were fed tap water and a veterinary diet free from any contaminants that might affect the study integrity. The National Research Council’s Guide for the Care and Use of Laboratory Animals was followed during the housing and care of the animals, and the study proposal was approved by the Faculty of Pharmacy’s Research Ethics Committee, Suez Canal University, Egypt, approval number: 202204MA3.

### 4.3. Study Design and Disease Induction

Forty male albino mice were divided randomly into five groups, each of eight mice. The groups were divided as follows:

1. Normal control group received the vehicle;
2. Positive control group was injected only SCOP (0.5 mg/kg, i.p.) [31];
3. CILO-treated group (10 mg/kg i.p.) [58] + SCOP (0.5 mg/kg, i.p.);
4. CGA-treated group (9 mg/kg i.p.) [59] + SCOP (0.5 mg/kg, i.p.);
5. Combination group: CILO (10 mg/kg i.p.) + CGA (9 mg/kg i.p.) + SCOP (0.5 mg/kg, i.p.).

**Figure 8:**
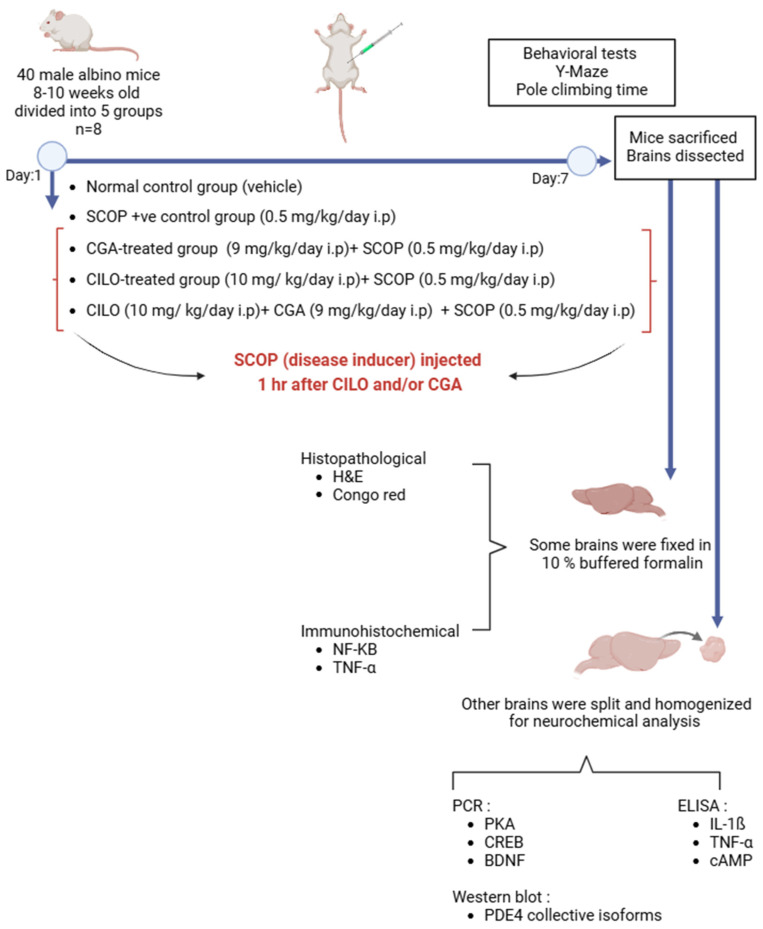
Flowchart of the study design. Forty male albino mice were divided into five groups (*n* = 8): normal control, SCOP, CILO, CGA and combination groups. The Y-maze and pole climbing tests were performed as behavioral tests. Histopathological examination and immuno-staining were conducted to detect the pathological changes of the brain. The levels of inflammatory markers and cAMP, together with the expression pattern of PDE4 and its related downstream target genes, were also determined.

SCOP (0.5 mg/kg, i.p.) was used as a model inducer and administered to all groups, except the normal control group. SCOP was administered one hour after pretreatment with CILO and/or CGA; 30 min afterward, behavioral tests were conducted [60]. Both SCOP administration and treatments were continued for seven consecutive days across all groups. The flowchart of this work is presented in Figure 8.

### 4.4. Behavioral Tests

#### 4.4.1. Y-Maze Test

The short-term memory of the experimental mice was assessed using the Y-maze test by observing spontaneous alternation. The Y-maze utilized in this investigation was of three identical arms that were established at a 120-degree angle to one another. Each of the Y-maze’s arms was given a different letter (A, B or C). Each mouse was put to the extremity of one arm and given 8 min to roam freely. Short-term memory was reflected in the spontaneous alternation of particular arm transition patterns (ABC, BCA, or CAB but not BAB, CAC or CBC). SA% was defined according to the formula:

$$SA\%=\frac{number\;of\;alterations\;(NO\;of\;SAP)}{Total\;arm\;entries-2}\times 100$$

where total arm entries = No. of entries to A + No. of entries to B + No. of entries to C [5].

#### 4.4.2. Pole Climbing Test

The main objective of this test was to evaluate the mice’s locomotor performance and coordination. In this experiment, a mouse is placed with its head up on the top of a 5 mm-diameter × 50 cm-long pole. The mouse then turns its head downward in an attempt to descend to the ground. The time required by the animal to bend his head downward and get down with the four paws on the ground was recorded [5].

### 4.5. Biochemical Assay

#### 4.5.1. Brain Tissue Preparation

The mice were sacrificed at the end of the experiment under light halothane anesthesia, and then, the entire brains were precisely dissected and promptly removed and rinsed. The brains were divided into portions for further analyses. The samples for histopathological investigation and immunohistochemical assessment were fixed in 10% buffered formalin, whereas the remaining samples were stored at −80 °C. Later, some of the frozen brain tissues were homogenized in PBS (pH 7.4) using TissueLyzer II (Qiagen, Hilden, Germany) for ELISA testing. The other frozen tissues were homogenized in a lysis buffer for qRT-PCR or in a radioimmunoprecipitation assay (RIPA) buffer for a Western blotting assay as according to specifications.

#### 4.5.2. Histopathological Examination

The fixed brain tissues were cleaned, alcohol-dried, xylene-cleared and paraffin-embedded. The tissue blocks were cut at a thickness of 4 μm. The sections of the tissues were put on microscopical slides and deparaffinized for staining. Some sections were stained with H & E to visualize the pathological changes. Other sections were stained with Congo red stain to visualize the precipitated amyloid plaques using a light electric microscope CX23 (Olympus Global, Tokyo, Japan). Evaluation of the degree of neurodegeneration and Aβ deposition in the brain was done using morphometric analysis.

#### 4.5.3. Immunohistochemistry Examination

IHC was used to assess the expression of NF-κB and TNF-α in brain tissue. For immunohistochemical staining, the deparaffinized slices from the brain tissue blocks were rehydrated and treated with proteinase K to extract the antigen. The activity of endogenous peroxidase was blocked by the addition of a hydrogen peroxide block (Thermo Scientific, Waltham, MA, USA). Tissue sections were treated with 10 mM citrate buffer and then microwaved for another 10 min to retrieve the antigen. The sections were then incubated overnight at 4 °C with either anti-NF-κB or anti-TNF-α (Dilution 1:100, Santa Cruz Biotechnology, Dallas, TX, USA). Slices were washed thrice in PBS for 5 min the next day; then, they were incubated with a secondary antibody for 30 min at room temperature before being conjugated with horseradish peroxidase (HRP) and detected with 0.025% diaminobenzidine (DAB). The images were captured by a light electric microscope CX23 (Olympus Global, Tokyo, Japan). The results were expressed as percentage of immune-positive cells at ×200 magnification [61].

#### 4.5.4. Determination of Inflammatory Cytokines and cAMP in Brain Tissue by ELISA

Using commercially available ELISA kits purchased from Elabscience^®^ (Houston, TX, USA), the levels of TNF-α (Cat No. E-EL-M3063) and IL-1β (Cat No. E-EL-M0037) were assessed in the brain tissue homogenate. On the other hand, the brain concentration of cAMP was measured using an ELK biotechnology^®^ ELISA kit (Cat No. ELK8116, St. Paul, MN, USA).

#### 4.5.5. Determination of PKA, CREB and BDNF Gene Expression by qRT-PCR

Total RNA was extracted from the brain tissue using the RNeasy Mini Kit (Cat No. 74104, QIAGEN, Hilden, Germany). Total RNA concentration was measured at 260 nm using the QIAxpert UV/VIS Spectrophotometer (QIAGEN, Hilden, Germany). Afterward, reverse transcription of the extracted RNA was performed to obtain complementary DNA (cDNA) using the RevertAid First Strand complementary DNA Synthesis Kit (Thermo Fisher Scientific, Waltham, MA, USA, Cat No. K1622). The real-time amplification step was performed by Maxima SYBR Green/ROX qPCR Master Mix (2X) (Cat No. K0221, Thermo Fisher Scientific, Waltham, MA, USA) using the Applied Biosystems^®^ 7500 Fast Real-Time PCR System (Thermo Fisher Scientific, Waltham, MA, USA). The used primers [6] were purchased from Macrogen (Seoul, Republic of Korea). The expression level of the targeted genes was quantified using the 2^−ΔΔCt^ method, and glyceraldehyde-3-phosphate dehydrogenase (GAPDH) was chosen as the endogenous control to normalize the samples variance.

#### 4.5.6. Determination of PDE4 Isoforms Expression by Western Blot Analysis

The Novagen^®^ Bicinchoninic Acid (BCA) Protein Assay Kit (Cat No. 71285, Merck, Rahway, NJ, USA) was used to measure the samples’ total protein concentrations. Then, the proteins were separated by 10–12% mini sodium dodecyl sulfate–poly acrylamide gel electrophoresis (SDS-PAGE) and transferred onto a 0.2 µm polyvinylidene fluoride (PVDF) membrane. Afterward, the membrane was blocked by 5% skimmed milk in Tris-buffered saline with Tween 20 (TBST) buffer solution at room temperature for one hour and incubated with polyclonal rabbit antibody against PDE4 total isoforms (all at a dilution of 1:500; Cat No. ELK1387, ELK Biotechnology, St. Paul, MN, USA) at 4 °C overnight. Following washing and incubation with HRP-conjugated secondary antibodies (Sigma Aldrich, Louis, MO, USA), the specific protein bands were detected using the electrochemiluminescence substrate (Thermo Fisher Scientific, Waltham, MA, USA) in strict adherence to the manufacturer’s guidance. Using the ChemiDock Gel documentation imaging system^™^ (BioRad, Contra Costa County, CA, USA), the protein signals were visualized and densitometric measurements were performed. The intensity of the signals was normalized against a GAPDH internal reference.

### 4.6. Protein–Protein Network Analysis

The protein–protein network analysis was created by the Cytoscape software v3.10.2. PKA, CREB, BDNF and PDE4 were selected as central proteins to establish the network.

### 4.7. Statistical Analysis

Data are represented as mean ± standard deviation (SD). Differences between groups were assessed using a one-way ANOVA test, and a post hoc analysis was conducted employing the Tukey multiple comparison test, using the IBM^®^ SPSS^®^ Statistics version 27 software (IBM Corp., Armonk, NY, USA). A *p*-value less than 0.05 was assumed to be significant. GraphPad Prism version 9.5.1 was used to plot the graphs.

## 5. Conclusions

**Figure 9:**
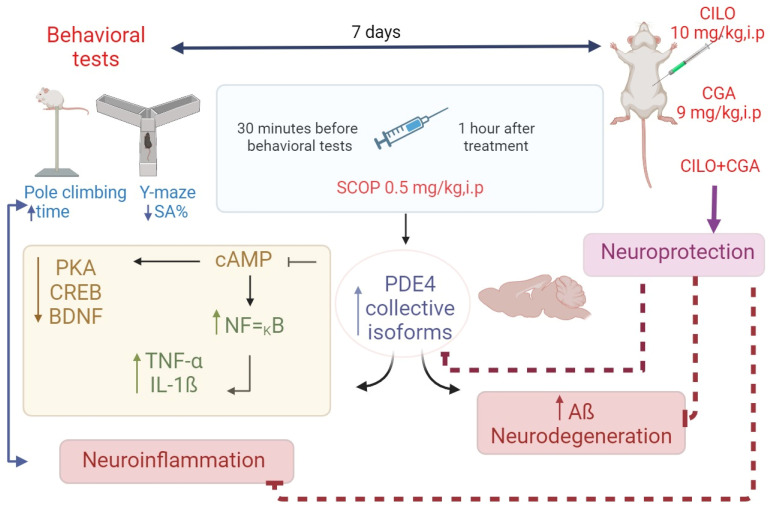
Illustration for the experimental design and the findings of the study.

Pretreatment of the mice with CILO remarkably amended the pathological changes induced by SCOP via modulation of the cAMP/PKA–CREB–BDNF signaling pathway, which includes the downstream targets of PDE4. This modulation concomitantly lowered the inflammatory response and reversed the deteriorated histopathological changes, which was reflected clearly in the behavioral performance of the mice. Co-administration of CGA with CILO caused further inhibition of PDE4, thereby augmenting its neuroprotection. These outcomes suggest that PDE4 inhibitors may represent potential neuroprotective agents. Our study and its findings are summarized in Figure 9.

## Data Availability

Data are available upon reasonable request.

## Abbreviations

The following abbreviations are used in this manuscript:

Aβ	Amyloid beta
PRKACA	Protein kinase A subunit alpha
CREBBP	CREB-binding protein
FOS	Fos proto-oncogene, AP-1 transcription factor subunit
ERK	Extracellular signal-regulated kinase
MAPK	Mitogen-activated protein kinase
AD	Alzheimer’s disease
ANOVA	Analysis of variance
BCA	Bicinchoninic Acid
BDNF	Brain-derived neurotrophic factor
BV	Blood vessels
cAMP	Cyclic adenosine monophosphate
cDNA	Complementary DNA
CGA	Chlorogenic acid
CILO	Cilomilast
CNS	Central nervous system
COX-2	Cyclooxygenase-2
CREB	cAMP response element-binding protein
DAB	Diaminobenzidine
DMSO	Dimethyl sulfoxide
ELISA	Enzyme-linked Immunosorbent Assay
EPAC1	Exchange Protein Activated by Cyclic-AMP1/2
ERK	Extracellular signal-regulated kinase
GAPDH	Glyceraldehyde-3-phosphate dehydrogenase
H & E	Hematoxylin and eosin
HRP	Horseradish peroxidase
IFN-γ	Interferon-γ
IHC	Immunohistochemistry
IL-17	Interleukin-17
IL-1β	Interleukin 1 beta
IL-6	Interleukin 6
LTP	Long-term potentiation
MAPK	Mitogen-activated protein kinase
NF-κB	Nuclear factor kappa B
NFTs	Neurofibrillary tangles
PBS	Phosphate buffered saline
PDE4	Phosphodiesterase-4
PKA	Protein kinase A
PVDF	Polyvinylidene fluoride
qRT-PCR	Quantitative real-time polymerase chain reaction
RIPA	Radioimmunoprecipitation assay
ROS	Reactive oxygen species
SA%	The percent of Spontaneous Alteration
SCOP	Scopolamine
SD	Standard deviation
SDS-PAGE	Sodium dodecyl sulfate–polyacrylamide gel electrophoresis
TNF-α	Tumor necrosis factor-alpha
